# Measuring pressure pain threshold in the cervical region of dizzy patients—The reliability of a pressure algometer

**DOI:** 10.1002/pri.1736

**Published:** 2018-08-07

**Authors:** Mari Kalland Knapstad, Stein Helge Glad Nordahl, Ingvill Fjell Naterstad, Tove Ask, Jan Sture Skouen, Frederik Kragerud Goplen

**Affiliations:** ^1^ Norwegian National Advisory Unit on Vestibular Disorders, Department of Otorhinolaryngology/Head and Neck Surgery Haukeland University Hospital Bergen Norway; ^2^ Department of Clinical Medicine University of Bergen Bergen Norway; ^3^ Department of Global Public Health and Primary Care University of Bergen Bergen Norway; ^4^ The Outpatient Spine Clinic, Department of Physical Medicine and Rehabilitation Haukeland University Hospital Bergen Norway; ^5^ The Outpatient Spine Clinic, Department of Physiotherapy Haukeland University Hospital Bergen Norway

**Keywords:** cervical, dizziness, neck pain, pressure algometer, pressure pain threshold

## Abstract

**Objectives:**

A tool for measuring neck pain in patients with dizziness is needed to further investigate the relationship between the two symptoms. The objective of this study was to examine the reliability and validity of a hand‐held pressure algometer in measuring pressure pain threshold (PPT) in different cervical regions of dizzy patients.

**Methods:**

PPT was measured at two bilateral standardized sites of the neck by a trained physiotherapist in 50 patients with dizziness. Intraclass correlation coefficients (ICC) were calculated for intrarater and test–retest reliability. Concurrent validity was assessed by measuring the association between PPT and the American College of Rheumatology (ACR) tender points at each site and with the numeric pain rating scale (NPRS).

**Results:**

Almost perfect intrarater (ICC = 0.815–0.940) and within‐session test–retest (ICC = 0.854–0.906) reliability was found between the measures. On each site, a low PPT predicted a positive ACR tender point at each site (OR = 0.864–0.922). Last, we found a statistical inverse relationship between the PPT and the NPRS (*R* = −0.52 to −0.66).

**Conclusion:**

The study shows that a pressure algometer is a reliable tool for measuring PPT in the neck of dizzy patients. Further, the PPT correlates significantly with other subjective measures of pain indicating that it may be a useful tool for further research.

## 1 INTRODUCTION

Concurrent neck pain is reported by up to one in three patients suffering from dizziness (Wilhelmsen, Ljunggren, Goplen, Eide, & Nordahl, 2009). Hypothetically, dizziness may lead to increased pressure sensitivity in the cervical region, due to a build‐up of muscular tension caused by fear of head movement (Furman & Jacob, 2001). However, the causal relationship between dizziness due to neck pain is controversial (Brandt & Bronstein, 2001). To further investigate the relationship between neck pain and dizziness, a reliable measurement tool is needed.

Self‐reported pain is commonly measured using a linear scale such as the numeric pain rating scale (NPRS). The interpretation of pain intensity on such a scale is complicated by the fact that self‐reported pain is affected not only by the level of organic tissue damage but also by psychosocial factors (Melzack, 2001). The American College of Rheumatology (ACR) tender points count is another measure, considered more as an identifier of chronic widespread pain (Limer, Nicholl, Thomson, & McBeth, 2008) and hypersensitivity (Harden et al., 2007). However, this measure is dependent on the patient's subjective experience of pain and the examiner's interpretation of the patient's behaviour withdrawing from stimulus, grimacing, etc (Harden et al. 2007). A pressure algometer is commonly used to quantify local pressure pain threshold (PPT) in different pain syndromes (Andersen, Petersen, Svendsen, & Gazerani, 2015; Walton et al., 2011). In theory, algometers could be useful for quantifying the PPT in the neck of dizzy patients to examine the relationship between the degree and the localization of neck pain and dizziness. Further, it could be useful in differentiating levels of pressure sensitivity in the upper and lower regions of the neck, because the mechanical properties differ, with the upper cervical spine being most mobile (Dutia, 1991). Previous studies of the intrarater reliability of hand‐held algometers in patients with neck pain have given conflicting results (Walton et al., 2011; Ylinen, Nykanen, Kautiainen, & Hakkinen, 2007). However, to our knowledge, no previous study has examined PPT using algometers in the neck of dizzy patients.

The aim of this study was to examine the reliability of a pressure algometer in different cervical regions in patients with dizziness, and its concurrent validity to other measures of pain. We examined the intrarater reliability of three consecutive measurements and the within‐session test–retest reliability of a pressure algometer at the upper and lower neck in patients with dizziness. In addition, we examined the association between PPT, ACR tender points, and NPRS.

## 2 METHODS

### 2.1 Subjects and setting

This study was conducted at a department of otorhinolaryngology, head, and neck surgery of a Norwegian university hospital. The department receives approximately 1,200 patients yearly referred due to dizziness or balance problems. Consecutive patients with dizziness aged 18–67 years were recruited from July to September 2017. Exclusion criteria were physical or language barriers in performing the tests or filling in the study questionnaires. The department is a quaternary referral centre for patients with vestibular schwannomas and for divers investigated for neuro‐otological disorders, and these two patient groups were excluded in order not to bias the results. The study protocol was approved by the Regional Committee for Medical and Health Research Ethics of South‐Eastern Norway (REK 2017/783). Participation was based on written informed consent.

### 2.2 ACR tender points

The ACR tender point count is a validated method for assessing widespread pain by applying pressure to nine defined bilateral tender points across the body (Segura‐Jimenez et al., 2014). The examiner used the thumb pad to apply a pressure, gradually increasing to a maximum of 4 kg, to each point. At each point, the patient signalled the presence or absence of pain with answering “yes” or discomfort/no pain by answering “no.” In the present study, only the upper four cervical sites were examined in order to evaluate the association with the PPT. The four sites were as follows: bilaterally suboccipital, 2 cm lateral to the spinous process of the axis (upper neck [UN]) and bilaterally at the anterior aspects of the intertransverse space at C5–C7 (lower neck [LN]). Each point was tested only once.

### 2.3 Pressure pain threshold

The PPT was measured by a trained physiotherapist, with a Wagner FDX‐25 device (Wagner Instruments, Greenwich, CT) on the upper four ACR tender points. The pressure algometer has linear response to force application between 0 and 1,300 kilopascal (kPa). The device has a 1‐cm^2^ round rubber tip. Prior to the study, the examiner practiced applying pressure at a rate of approximately 50 kPa/s. Continuous ability to reach 250 kPa in 5 s was considered applicable for testing patients. PPT was assessed with the patient in supine position with the arms down alongside the body. The PPT of the participants was assessed by the same examiner. The algometer maintained its peak value, so the examiner could be blinded to the display score while applying pressure. The patient was told to immediately state when the pressure sensation changed into a pain sensation, at which time, the pressure was stopped, and the score was noted. Three measurements (PPT1, PPT2, and PPT3) were recorded at the previously described four cervical sites (UN and LN), starting left at the suboccipital site and ending right on the intertransverse space at C5–C6. Approximately 30 s separated measurement of the same site. After approximately 30 min, two more measurements (PPT4 and PPT5) at each site were recorded using the same procedure.

### 2.4 Numeric pain rating scale

Patients reporting neck pain were asked to rate their neck pain during the two last weeks on an 11‐point scale. The NPRS has previously shown adequate reliability and validity in patients with chronic pain conditions, including neck pain (Childs, Piva, & Fritz, 2005; Cleland, Childs, & Whitman, 2008; Hawker, Mian, Kendzerska, & French, 2011). The pain is rated from 0 to 10 where 0 represents *no pain* and 10 represents the *worst imaginable pain*.

### 2.5 Procedure sequence

The procedure started with the first three PPT assessments. Second, the ACR tender points were examined 5 min after the PPT measurements. The retest of PPT was assessed 30 min after the first assessment. After the assessment, the patients filled in the questionnaires confidentially and handed it to a study nurse so that the examiner was blinded as to whether or not the patient reported neck pain. The NPRS was filled in by patients answering “yes” to the question “have you experienced neck pain during the last 14 days.”

### 2.6 Statistical methods

After visual inspection of histograms, the data were considered satisfactory for parametric analysis. An alpha value of 0.05 was selected. Based on the estimations of Donner and Eliasziw (1987), and the previous results of Ylinen et al. (2007) and Sterling, Jull, Carlsson, and Crommert (2002), we calculated the required number of subjects to *n* = 50 to reach 80% power with an alpha level of 0.05 (Donner & Eliasziw, 1987). Descriptive data were reported as mean and standard deviation. Two types of reliability were assessed: relative and absolute. Intrarater reliability and test–retest relative reliability were assessed using the one‐way random intraclass correlation coefficient (ICC 1.1) model. The ICC 1.1 was assessed between all measures. In order to detect systematic errors, the measurements were additionally analysed using two‐way mixed ICC model (3.1; Weir, 2005). Benchmark for ICC values was set according to Landis and Koch (1977): <0.4 is considered unacceptable, 0.41–0.60 moderate, 0.61–0.80 substantial, and 0.81–1.00 almost perfect agreement. Absolute reliability was assessed using the within‐subject standard deviation (S_w_) as described by Bland & Altman (1996). Although ICC values measure relative reliability, the S_w_ indicates absolute reliability, meaning true level of agreement. It is reported in the same units as the algometer (kPa). The difference between a subject's measurement and the true value would be expected to be less than 1.96 S_w_ for 95% of observations. The minimal detectable change (MDC) was calculated at the 90% level with the formula S_w_ × √2 × 1.64 (Walton et al., 2011). It is used to estimate a score in which the rater is 90% confident that a true change has occurred beyond the measurement error. The mean PPT at the four different sites was calculated between the baseline measures with highest reliability and used in further analysis. Binary logistic regression was performed to determine if the PPT could predict a positive outcome to its equivalent ACR tender point in the neck. Linear and multiple linear regressions were used to examine association between PPT and NPRS in patients reporting neck pain. The PPT for each site was analysed in separate regressions, due to multicollinearity. Gender and age were used as covariates. The presence of self‐reported neck pain over the last 14 days was used as a grouping variable (yes/no neck pain). Data were analysed using SPSS version 24 for Windows (Statistical Package for the Social Sciences, SPSS Inc., Chicago, IL).

## 3 RESULTS

The study included 50 adult subjects aged 24–67 years (mean age 46 and standard deviation 12 years). There were 19 males (38%) and 31 females (62%). Neck pain was reported by 22 participants (44%). Descriptive statistics are reported in Table 1.

**Table 1:** Measurements of pressure pain threshold (kPa) in upper and lower neck, tender points, and numeric pain rating scale

Variable	Overall (*N* = 50)	No neck pain (*N* = 28)	Neck pain (*N* = 22)
UN left, mean (*SD*)^a^	239 (117)	260 (114)	211 (118)
UN right, mean (*SD*)^a^	224 (106)	238 (100)	207 (60)
LN left, mean (*SD*)^a^	177 (82)	193 (88)	155 (68)
LN right, mean (*SD*)^a^	167 (74)	185 (96)	141 (60)
ACR tender points neck, mean (*SD*)	2.0 (1.4)	1.7 (1.3)	2.5 (1.5)
NPRS, mean (*SD*)			4.4 (1.7)

*Note*. ACR: American College of Rheumatology; kPa: kilopascal; LN: lower neck; NPRS: numeric pain rating scale; PPT: pressure point threshold; UN: upper neck.

a Mean of measurements 2 and 3.

### 3.1 Reliability

Intrarater and test–retest reliability was examined by ICC 1.1. We found that the highest intrarater reliability was obtained between PPT2 and PPT3 in the first session at each site, disregarding the first measurement. There was an almost perfect reliability on all sites between these two measures. The test–retest showed an almost perfect reliability between PPT3 of the first test session and PPT5 at the retest session. The reliability was approximately the same irrespective of whether or not the patients reported neck pain. Performing the same analysis with ICC 1.3 showed no deviations in ICC values, indicating that no systematic errors were present. The ICC values, S_w_, and MDC for the intrarater and test–retest reliability are presented in Table 2.

**Table 2:** Intrarater reliability and test–retest reliability of pressure pain threshold at four cervical sites in 50 patients with dizziness

Test	Measure	ICC	95% CI	S_w_ (kPa)	±1.96 S_w_	MDC
Intrarater reliability (PPT2 and PPT3)^a^	Upper neck left	0.940	0.897, 0.965	29.1	±57.0	67.5
	Upper neck right	0.916	0.857, 0.951	31.3	±61.3	72.6
	Lower neck left	0.815	0.692, 0.889	37.1	±72.7	86.1
	Lower neck right	0.935	0.888, 0.962	19.2	±37.6	44.5
Test–retest reliability (PPT3 and PPT5)^b^	Upper neck left	0.906	0.841, 0.945	36.8	±72.1	85.4
	Upper neck right	0.876	0.793, 0.928	38.0	±74.5	88.2
	Lower neck left	0.854	0.757, 0.914	33.5	±65.7	77.7
	Lower neck right	0.862	0.770, 0.919	29.9	±58.6	69.4

*Note*. CI: confidence interval; ICC: intraclass correlation; MDC: minimal detectable change; PPT: pressure pain threshold; S_w_: within‐subject standard deviation.

a Between the second and third measures.

b Between the third and fifth measures.

### 3.2 Concurrent validity

As PPT2 and PPT3 had the highest reliability, the mean of these two measures was used to examine relationships with ACR tender points and NPRS. Using binary logistic regression, PPT in each of the four sites predicted a positive outcome at the equivalent ACR tender point: UN left, OR = 0.918, 95% CI [0.859, 0.981], *p* = 0.011, UN right OR = 0.922, 95% CI [0.862, 0.986], *p* = 0.018, LN left OR = 0.864, 95% CI [0.774, 0.964], *p* = 0.009, LN right OR = 0.874, 95% CI [0.789, 0.968], *p* = 0.010. The analysis adjusted for age and gender is shown in Table 3.

**Table 3:** Adjusted logistic regression between tender points and pressure pain threshold (*n* = 50)

	TP UN left			TP UN right			TP LN left			TP LN right		
Model	*p*	*X* ^2^	*R* ^2^ a	*p*	*X* ^2^	*R* ^2^ a	*p*	*X* ^2^	*R* ^2^ a	*p*	*X* ^2^	*R* ^2^ a
	0.02	10.25	0.15	0.04	8.22	0.12	0.003	13.8	0.19	0.02	9.44	0.14
Var	*p*	OR	CI	*p*	OR	CI	*p*	OR	CI	*p*	OR	CI
PPT, (kPa)	0.02	0.92	0.86, 0.98	0.02	0.92	0.085, 0.98	0.006	0.84	0.74, 0.95	0.015	0.87	0.77, 0.97
Age (years)	0.35	1.02	0.46, 7.25	0.31	1.02	0.97, 1.08	0.11	1.05	0.99, 1.11	0.55	1.02	0.96, 1.07
Gender (male)	0.39	1.83	0.05, 18.13	0.83	1.15	0.31, 4.20	0.58	0.67	0.15, 2.84	0.93	0.94	0.24, 3.78

*Note*. CI: confidence intervals; kPa: kilopascal; LN: lower neck; *p*: *p*‐value; PPT: pressure pain threshold; TP: the American College of Rheumatology tender points; UN: upper neck; Var: variables; *X* ^2^: mode chi‐square.

a Cox and Snell pseudo *R* ^2^.

Using linear regression, there was a significant association between PPT and NPRS at UN left (*R* = −0.62, *p* = 0.002), UN right (*R* = −0.66, *p* = 0.001), LN left (*R* = −0.63, *p* = 0.002), and LN right (*R* = −0.52, *p* = 0.01). The adjusted analysis with multiple linear regression is shown in Table 4. Gender and age did not significantly affect the associations.

**Table 4:** Multiple linear regressions between numeric pain rating scale and pressure pain threshold at the different measurement sites (*n* = 22)

Site	UN left			UN right			LN left			LN right		
Model	*p*	*R* ^2^		*p*	*R* ^2^		*p*	*R* ^2^		*p*	*R* ^2^	
	0.026	0.39		0.01	0.44		0.02	0.41		0.10	0.28	
Variable	*B*	*SE*	*p*	*B*	*SE*	*p*	*B*	*SE*	*p*	*B*	*SE*	*p*
PPT (kPa)	−0.09	0.28	0.005	−0.10	0.03	0.002	−0.15	0.05	0.004	−0.14	0.06	0.027
Age (years)	0.01	0.02	0.615	0.01	0.02	0.682	0.01	0.02	0.690	0.01	0.03	0.793
Gender (male)	0.017	0.69	0.918	−0.21	0.69	0.764	0.04	0.05	0.958	0.26	0.74	0.728

*Note*. kPa: kilopascal; LN: lower neck; *p*: *p*‐value; PPT: pressure pain threshold; *R* ^2^: model *R* squared; TP: the American College of Rheumatology tender points; UN: upper neck.

## 4 DISCUSSION

We examined the intrarater and test–retest reliability of a pressure algometer in testing PPT in the upper and lower neck of dizzy patients. Further, we examined the concurrent validity of PPT to the four upper ACR tender points and NPRS. We found high reliability for all PPT measures, in addition to significant association between PPT and ACR tender points at all four sites and to NPRS scores.

Our study found an almost perfect reliability of the pressure algometer at all four test sites under both the intrarater and test–retest conditions. Few studies have previously examined the reliability of the PPT at the sites measured in this study. Still, a high test–retest reliability (ICC 0.84–0.93) has been found at the suboccipital site in women with chronic neck pain (Ylinen et al., 2007) and the vertebral area C5–C6 (ICC 0.88–0.92) in patients with chronic neck pain (Sterling et al., 2002). To our knowledge, no studies have previously investigated PPT in the neck of dizzy patients.

The highest intrarater reliability was found between the second and third measurements, and the highest test–retest reliability was found between the last two measurements. Previous studies using other sites show somewhat conflicting results with regard to ICC comparison between trials (Balaguier, Madeleine, & Vuillerme, 2016; Walton et al., 2011). Balaguier et al. (2016) found high reliability between all three measures at sites in the lower back. Walton et al. (2011) found high reliability between measures 2 and 3 in the upper fibres of trapezius, and in agreement with our findings, reporting that the first measurement was less consistent. In this study, the S_w_ compared with the overall means indicate that measurement properties were acceptable for intrarater reliability, with the S_w_ ranging from 19.2 to 37.0 kPa, and for test–retest; ranging from 29.9 to 38.0 at the different sites. No previous studies have examined S_w_ at these sites, making the interpretation and comparison difficult. The reliability was similar regardless of whether or not patients reported neck pain, indicating that the presence of symptoms does not adversely affect the reliability of the results. The MDC for the intrarater session ranged from 44.5 to 86.1 kPa and from 69.4 to 88.2 kPa in the test–retest session at the different sites. The differences between the two sessions were smaller in our study, compared with the study by Walton et al. (2011), which found an MDC of 42.7 kPa at the intrarater session and 113.4 kPa in the test–retest session in the upper fibres of trapezius muscle in patients with neck pain. The relatively small difference in mean PPT between the two groups with and without pain might be due to the fact that they were not matched. Additionally, the PPT was measured at standardized sites and not necessarily the sites that the patient considered as most painful.

The ACR tender point count is a well‐known clinical examination often used in patients with widespread pain (Limer et al., 2008). We found that a low PPT predicted a positive tender point at each of the four corresponding cervical sites. The relationship between PPT and ACR tender points has been examined previously with conflicting results (Tastekin, Uzunca, Sut, Birtane, & Mercimek, 2010; Wolfe, 1997). However, in this study, we only examined the tender points in the upper and lower cervical regions. In theory, the algometer has the potential for higher precision and reliability because it measures the PPT as a continuous variable rather than as a binary outcome as in each of the ACR tender points.

There was a significant inverse association between PPT and NPRS in the patients reporting neck pain. These findings are interesting because PPT is influenced by both the examiner's execution of the test and the patient's interpretation, whereas the NPRS is only affected by the patient's own interpretation of pain intensity. Thus, our results suggest that the PPT associates with the patient's own experience of pain, which is an important observation when considering the use of PPT in further studies. The PPT has the additional advantage to being able to differentiate regions in the neck, which is difficult with the NPRS. The associations between PPT, ACR tender points, and NPRS were not significantly influenced by age or gender. Males and females have been found to differ with regard to pain thresholds (Fillingim, King, Ribeiro‐Dasilva, Rahim‐Williams, & Riley, 2009), with females reporting lower PPT compared with men (Fischer, 1987; Park, Kim, Park, Kim, & Jang, 2011). However, gender did not affect the outcome in the present study.

A potential limitation of the study was that the time interval between the test–retest conditions was only 30 min and that there was only one examiner. The result cannot be generalized to longer time intervals or tests made by different examiners. The strength of this study was the sufficient sample size and that there were no systematic errors in the measurements, making the results more applicable. Using the ACR tender points as measurement sites for PPT enabled us not only to examine the relationship between the two methods but also to measure pain at standardized sites in different regions of the neck.

### 4.1 Implication for physiotherapy practice

This study shows that a pressure algometer can be used to reliably measure PPT in both the upper and lower cervical regions in patients suffering from dizziness, indicating that it may be a useful tool for further research on the level of pain threshold in the neck in this patient group. The results may contribute to future studies of the relationship between neck pain and dizziness.
